# A Corpus-Based Study on the Pragmatic Use of the ba Construction in Early Childhood Mandarin Chinese

**DOI:** 10.3389/fpsyg.2020.607818

**Published:** 2021-01-15

**Authors:** Linda Tsung, Yang Frank Gong

**Affiliations:** ^1^Faculty of Arts and Social Sciences, The University of Sydney, Darlington, NSW, Australia; ^2^Faulty of Education, University of Macau, Taipa, China

**Keywords:** preschool children, Mandarin Chinese, ba construction, corpus-based study, language development

## Abstract

This article reports on an inquiry that investigated the development of ba constructions in early childhood Mandarin. All cases of ba construction were extracted from the Early Childhood Mandarin Corpus collected from 168 preschoolers aged 2;6, 3;6, 4;6, and 5;6 (year; month; Li and Tse, 2011). Early Childhood Mandarin Corpus, University of Hong Kong. Data analysis indicated that: (1) Mandarin-speaking children produced a repertoire of 11 types of ba construction, and the children in the youngest age group (age 2;6) were able to produce six types of them; (2) children at 4 years old (age 4;6) experienced a critical developmental period of pragmatic use, and at 5 years old (age 5;6) they had attained cognitive and linguistic maturity in understanding the semantic and syntactic features of ba constructions; and (3) there was a significant age effect on the production of three types of ba construction, but no significant association between the children’s gender and their production of ba constructions. These findings offer fresh insights into understanding Chinese children’s innate capacity to understand the co-occurrence constraints concerning the syntactic, semantic and verb features inherent in ba construction, and their developmental ability to denote telic events by resorting to the appropriate ba sentence patterns.

## Introduction

The ba construction is frequently used by native speakers of Mandarin Chinese. Different from canonical “Subjective-VP-Objective (SVO)” sentences in Mandarin Chinese, the ba construction takes the form of “ba-Object-VP.” It is seen as a unique grammatical pattern by many linguists (Wang, 1945; Liu, 1997; Ziegeler, 2000; Xu, 2011) and researchers of child language development (Li et al., 1990; Deng et al., 2018). At the same time, due to its structural peculiarity there is no syntactic pattern corresponding to the ba construction in other languages, especially Western languages such as English (Wang, 1945; van der Lee, 2018). The construction presents a range of difficulties and challenges for both L1 and L2 Chinese language learners in terms of its acquisition (e.g., Wen, 2012; Yi, 2014). For this reason, there is a pressing need for researchers to investigate Chinese language learners’ developmental patterns related to the acquisition of the ba construction (Ma et al., 2017; Gong et al., 2018, 2020a,b).

Previous research has documented children’s production of the ba construction using different research methods, such as contrived topic-specific experiments (e.g., Li et al., 1990; Gong, 2007) and case studies (e.g., Yang and Xiao, 2008; Chang and Zheng, 2017). These studies have mainly analyzed the overall trends in children’s acquisition of ba sentences. Specifically, the ba construction has been reported to emerge at approximately age 2;0 (year; month) and develop significantly by around age 4;6. However, the ba construction can be classified into various categories or types in the actual discourse environment, and little attention has been paid to the developmental patterns associated with specific syntactic forms. Moreover, it is crucial to expand sample sizes and collect naturalistic language materials from children’s authentic utterances in order to understand the natural use of ba sentences in their language production. Relatively few studies have adopted authentic language data, such as a corpus of spontaneous speech from different participants, to explore Mandarin-speaking preschoolers’ acquisition of the ba construction.

In addition, previous research has noted little about the impact of age or gender on acquiring the ba construction, and thus has been unable to completely depict the multifaceted complexity of child language acquisition (e.g., Yi, 2014; Chang and Zheng, 2017). Specifically, the developmental pattern of and gender differences in the acquisition and use of syntactic, semantic and verb features inherent in ba sentences have not been examined to any great extent.

To address the research gaps described above, the present study examined the developmental order of the acquisition of different types of ba construction by Mandarin-speaking children, using naturalistic language data from the Early Childhood Mandarin Corpus (ECMC; Li and Tse, 2011).

## Literature Review

### Lexical and Syntactic Aspects of the ba Construction

The ba construction in Chinese is unique in its sentence structure, which occurs as the pattern of “NP1 ba NP2 VP.” As described by many Chinese linguists, the ba construction has abstract meanings, such as “disposal,” “causation,” and “displacement” (e.g., Wang, 1945; Liu, 1997; Ye et al., 2007; Xu, 2011). The use of ba is not always possible, and is subject to several constraints. According to Huang et al. (2009), the ba construction is used when “an object is affected, dealt with, or disposed of” (p. 154). That is, the object of a ba sentence should always be “affected” or “influenced” by the verb phrase of the sentence. In the following example, it is evident that the object (the clothes) is affected by the verb (to wash), since the clothes have been washed clean.

Example 1:
他把我的衣服洗干净了
Ta ba wode yifu xi gangjing le (van der Lee, 2018)
He ba my clothes wash clean-ASP
He washed my clothes

Another constraint relates to the semantics of sentences using the ba construction. According to Wiedenhof (2012), the object in a ba sentence is always definite or specific (also see Li, 1993; Ye et al., 2007). In other words, the object should be known from the discourse context, as in the following example provided by Liu (2007, p. 650). At the same time, the verb in a ba sentence should be a delimiting one. In the example sentence, the verb 送 (song, send) should be converted into a delimiting predicate 送走 (songzou, send away) before it can appear, which denotes a resultative meaning. In this sense, Liu (1997) proposed that ba is compatible with predicates that describe delimited events and the ba construction has two inherent properties: boundedness, and specificity.

Example 2:
我想把三个学生送走
Wo xiang ba sange xuesheng songzou (Liu, 2007)
I want ba three-CL students send away
I want to send away three (particular) students

Besides the semantic requirements above, there is also a crucial requirement regarding the verb of a ba sentence. The verb always needs to be transitive and complex in order for a ba sentence to be grammatical (Ye et al., 2007; Wen, 2012; van der Lee, 2018). This means that the verb can never stand alone, but always needs to be accompanied by another element, such as an aspect marker (e.g., 了, le, perfective marker; 着, zhe, durative marker; and 给, gei, dative marker), a verb reduplication, or a verbal quantifier, to indicate the effects of the verb on the rest of the sentence. In this regard, Sun (1995) noted that “ba functions to mark a high degree of transitivity” (p. 159). The post-verbal complement typically conveys telic, perfective, and resultative meanings. The following ba sentence (Example 3) would sound incomplete or would not be grammatically correct if there was no perfective aspect marker 了 (le), especially as a past-time reference 昨天 (zuotian, yesterday) is intended.

Example 3:
我昨天把那辆车卖了
Wo zuotian ba na liang che mai-le
I yesterday ba that CL car sell-ASP
I sold that car yesterday

### Pragmatic Aspect of the ba Construction

While semantics relate to the original or ordinary meanings of a word in a language and words that can only be used in certain structures, the pragmatic use of a word can demonstrate various features in a natural discourse context (Griffiths, 2006). In Mandarin Chinese, the ba construction is widely used to present events with temporal properties such as “telicity, boundedness and perceptivity” (Deng et al., 2018, p. 244), and has many different pragmatic meanings. Thus, young children need to acquire all the lexical, syntactic, and pragmatic meanings of the ba construction in Mandarin Chinese to express relevant sentences appropriately. In this sense, examining children’s pragmatic use of ba sentences will shed light on the development of temporal understanding and actual expressions in the early years.

However, linguists and researchers have espoused different views on the pragmatics of ba construction in Mandarin Chinese. For instance, Liu (1997) proposed that the ba predicate expresses bounded events, and classified the authentic use environment of ba into nine types (see Supplementary Appendix 1). In this framework, the high transitivity of the ba construction seemed to be omitted, and thus no “给-gei + verb/noun” cases emerged. However, cross-linguistic findings on children’s language acquisition and use suggest that transitive events with regard to object transfer and physical manipulation are usually expressed by young children (e.g., Bavin, 1995). Based on a self-built corpus of language generated by Mandarin Chinese children (from age 1;2 to 5;0), Wang (2012) investigated children’s acquisition of ba construction and classified it into six categories and 13 sub-categories (see Supplementary Appendix 2). In Mandarin Chinese the perfective viewpoint is mainly marked by 了-le and the durative by 着-zhe, and therefore a category like “V + 了-le/着-zhe” should be further distinguished in any examination of Mandarin-speaking preschoolers’ productive speech. Li et al. (1990) examined the acquisition and use of ba sentences among 70 Mandarin-speaking children (aged 2;0, 2;6, 3;0, 3;6, 4;0, 4;6, and 5;0) and divided the pragmatic environments into nine types and 17 sub-types (see Supplementary Appendix 3). While the resultativity of ba constructions was to some extent addressed in this framework, the wide use of resultative verb complements in ba sentences seemed to be omitted. Thus, it was necessary to create a new category for “V + resultative verb complement” in a new analytical framework to investigate young children’s language materials. On the basis of understanding the constituent after the verb (post-verbal elements) behind ba, Lü (1984) proposed a thirteen-case framework with five major classes (see Supplementary Appendix 4). Even through the verb complements behind ba were divided into different categories in light of a syntactic criterion (complexity of verb forms), aspectual properties of ba constructions such as the perfective aspect marker 了-le and the durative aspect marker 着-zhe did not emerge in this framework. Hence, a new analytical framework was required, to provide more information about the aspectual properties of ba constructions in children’s Mandarin Chinese.

Comparing the existing frameworks, it is evident that the ba construction has diverse pragmatic meanings according to the semantic aspect of the element behind ba in the same collative structure. Each of the extant frameworks proposed by different scholars (e.g., Lü, 1984; Li et al., 1990; Liu, 1997; Wang, 2012) has their own strengths and weaknesses. At the same time, we assume that young children may encounter difficulties in understanding and acquiring all the specific pragmatic meanings and producing the irregular forms of the ba construction. Therefore, in order to elicit all possible natural utterances using a ba construction from the corpus (Li and Tse, 2011), it was decided that a compromised or combined framework might be more appropriate and more practical, developed by comparing and synthesizing the four frameworks described above. Specifically, the following 11-category framework is proposed to investigate the pragmatics of ba construction in the present study (see Table 1).

**TABLE 1:** The framework adopted in the present study.

Category	Example
1) V + resultative verb complement	你把问题看清楚 Ni ba wenti kan qingchu You ba question read clear You read the questions so that they are clear
2) V + (_一_-yi) + V	把车修（一）修 Ba che xiu-yi-xiu Ba car fix-one-fix Fix the car a little
3) V + noun (possessive, person, resultative, and partitive)	把嘴扎流血 Ba zui zha liuxue Ba mouth stab bleed Stab the mouth to bleed 我把字典给王先生 Wo ba zidian gei Mr. Wang I ba dictionary give Mr. Wang I gave the dictionary to Mr. Wang 把这个染上颜色 Ba zhe ge ran shang yansey Ba this dye up color Dye this 把一个南京城走了大半个 Ba yige nanjingcheng zou-le da bange Ba one Nanjing city go-ASP big half Walk through half of Nanjing City
4) V + quantified phrase	他把电影看两遍 Ta ba dianying kan liangbian He ba movie watch twice He watches the movie twice
5) V + 了-le	他把苹果吃了 Ta ba pingguo chi-le He ba apple eat-ASP He ate the apple
6) V + 着-zhe	把这两个都拿着 Ba liangge dou na-zhe Ba two both hold-ASP Hold both of them
7) V + 得-de	他把我哭得心烦 Ta ba wo ku de xinfan He ba I cry DE heart-disturbed He cried so much that I became disturbed
8) V + 在-zai/到-dao + locative	把它放在嘴里 Ba ta fang zai zuili Ba it put ZAI mouth-in Put it into (the) mouth
9) 给-gei + verb/noun	把那个给我 Ba nage gei wo Ba that GEI me Give that to me 把我给累死了 Ba wo gei lei si-le Ba me GEI exhaust die-ASP Exhaust me to death
10) 成-cheng + noun	把它弄成面包 Ba ta nong cheng mianbao Ba it make CHENG bread Make it into bread
11) Adv + verb	我把桌子往屋里搬 Wo ba zhuozi wang wuli ban I ba table toward room-in move I was moving the table into the room

The present framework, adapted from previous work, was designed to cover more of the pragmatic environments where the ba construction occurs. This was mainly because the participants in the present study are preschoolers, who are in an early stage of developing their cognitive and linguistic ability to understand and express temporal concepts. Therefore, they may present more irregularity regarding the use of ba constructions.

### Acquisition of the ba Construction

Over the past three decades, a number of scholars have investigated how Mandarin-speaking children acquire the ba construction. Research evidence points to two major topics: children’s production stages, and sentence patterns. For example, Li et al. (1990) found that preschoolers’ production of ba sentences occurred at around age 2;0 and accounted for 90% of adults’ ba construction types at age 4;6. They classified its environments into nine major categories (17 sub-categories), and further noted that “ba + V + verb/adjective,” “ba + V + directional verb,” and “ba + V + 在-zai/到-dao + locative” were the three most frequently used forms of ba construction. In a similar vein, Wang (2012) adopted language data from a self-built corpus of Mandarin-speaking children aged from 1;2 to 5;0 (sample size unclear). This research suggested that the participants started producing ba sentences at approximately age 2;0 and divided their productions into six types, including 13 sub-types. Moreover, it was found that four ba construction structures were used most often: (1) “ba + V + directional verb,” (2) “ba + V + verb,” (3) “ba + V + 在-zai/到-dao + locative,” and (4) “ba + V + 了-le/着-zhe.” Chang and Zheng (2017) investigated the development of ba construction with a Mandarin-speaking boy over a period of 35 months, and indicated that “ba + V + resultative verb complement” occurred most often. Gong (2007) conducted experimental research with 30 preschoolers aged from 4;4 to 5;4 and reported that their syntactic judgment accuracy did not increase with age. In an investigation of the productive speech of ninety-nine Mandarin-speaking children, Li (1993) reported that the participants showed awareness of the co-occurrence restrictions inherent in ba sentences and a good command of the use of ba construction from age 3.

While there seems to be agreement in terms of the overall developmental trend of children’s acquisition of ba constructions at different ages, their production patterns in different specific structures are still little known and underexplored. At the same time, the relationships between children’s production of ba sentences and their age and gender have received limited attention. Accordingly, the present research aims to address the following two questions:

RQ1: What are the repertoires of Mandarin-speaking children’s acquisition of ba constructions during early childhood?
RQ2: What developmental patterns can be observed in Mandarin-speaking children’s acquisition of ba constructions during early childhood?

## Methodology

### Participants and the Corpus

The Early Childhood Mandarin Corpus (Li and Tse, 2011) used in the present research represents the utterances produced by 168 Mandarin-speaking preschoolers. This sample contained children from four age groups (ages 2;6, 3;6, 4;6, and 5;6), with 21 girls and 21 boys in each age group. They were randomly sampled from eight preschools located in the four major districts of Beijing, China: Haidian, Chaoyang, Dongcheng, and Xicheng. All the participating children were native speakers of Mandarin Chinese, and their parents and teachers also spoke Mandarin as their native language at home and in the preschool, respectively. The corpus consisted of these children’s natural utterances during 30-min free-play sessions in pairs. In total, 42 h of conversations were collected.

### Communication Task and Data Transcription

Participants of the same age were randomly paired (girl/girl, boy/boy, or girl/boy). They were encouraged to talk with each other while playing in a play corner for 30 min. The play corner was furnished with a set of toys, including faux food and fruits, cooking materials, furniture and electrical appliances, vehicles, and hospital materials. The participants’ conversations during playtime were videotaped using a high-definition digital camera. Two trained research assistants observed their activities but did not intervene during the sessions.

All the conversations were transcribed verbatim into Chinese and double-checked for accuracy by experienced research assistants and one researcher. It should be noted that non-lexical fillers such as “uh” and other vocalizations (e.g., laughter) were also included during the transcription (Tse et al., 2012). The final Chinese script was segmented into individual utterances. All the ba sentences in the utterances were first identified by the research assistants and the researcher and then reviewed and assessed by a Chinese linguist interested in modern Chinese grammar and the ba construction.

### Coding of ba Sentences

The eleven-type pragmatic framework proposed above was used to code all the expressions using ba. Two authors of this paper analyzed and coded all the ba sentences in the corpus and their mutual agreement was 95.1%, indicating excellent inter-coder reliability. In addition, some unrecognizable expressions without lexical, syntactic or pragmatic meaning (e.g., 別把你弄, bie ba ni nong, do not ba you make) were manually excluded from the dataset. In total, 435 ba sentences from 670 natural utterances with ba were identified and coded.

In the coding stage, four ba sentences (0.92%) were found to be not in line with type 5 or 6 of the initial framework, and thus we revised type 6 to be “V + 了-le/着-zhe + verb” to cover these sentences.

## Results

A total of 435 ba sentences was elicited from the corpus, produced by 97 participating children (age 2;6 = 17; age 3;6 = 15; age 4;6 = 27; and age 5;6 = 38. Girls = 48; boys = 49), which accounted for 57.7% of the original corpus. Overall, each participant produced 4.8 cases during their half-hour free-play sessions. Specifically, the participants in the four age groups produced 28, 28, 136, and 243 cases of ba construction, respectively. On average, each child in the 2;6 age group uttered 1.6 ba sentences, in the 3;6 age group 1.9 ba sentences, in the 4;6 age group 5.0 sentences, and in the 5;6 age group 6.4 ba sentences, in an ascending pattern.

### The Repertoire of Mandarin-Speaking Children’s Acquisition of the ba Construction

Descriptive analysis was conducted to examine all the participants’ production of ba constructions. Overall, 11 different types of ba construction were identified from the participants’ spontaneous speech. In particular, as shown in Table 2, the most frequently used types of ba construction were type 1 (141), 9 (82), and type 3 (69), which constituted 32.4, 18.9, and 15.9% of all occurrences in the corpus, respectively. The least used three types were type 6 (4), 4 (2), and 7 (2), with each representing only 0.9, 0.5, and 0.5% of all occurrences, respectively.

**TABLE 2:** The distribution of ba constructions across different age groups.

Types of ba constructions	Age groups				Total number and percentage of pragmatic use (435; 100%)	Ranking

	Age 2;6	Age 3;6	Age 4;6	Age 5;6		
			
	Number and percentage of pragmatic use (28; 6.4%)	Number and percentage of pragmatic use (28; 6.4%)	Number and percentage of pragmatic use (136; 31.3%)	Number and percentage of pragmatic use (243; 55.9%)		
1. V + resultative verb complement	18 (64.3%)	11 (39.3%)	40 (29.4%)	72 (29.6%)	141 (32.4%)	1
2. V + (_一_-yi) + V	1 (3.6%)	0 (0%)	10 (7.4%)	6 (2.5%)	17 (3.9%)	7
3. V + noun (possessive, person, resultative, and partitive)	4 (14.3%)	5 (17.9%)	21 (15.4%)	39 (16.0%)	69 (15.9%)	3
4. V + quantified phrase	0 (0%)	1 (3.6%)	1 (0.7%)	0 (0%)	2 (0.5%)	10
5. V + 了-le	0 (0%)	1 (3.6%)	9 (6.6%)	17 (7.0%)	27 (6.2%)	6
6. V + 了-le/著-zhe + verb	0 (0%)	0 (0%)	0 (0%)	4 (1.6%)	4 (0.9%)	9
7. V + 得-de	0 (0%)	0 (0%)	1 (0.7%)	1 (0.4%)	2 (0.5%)	10
8. V + 在-zai/到-dao + locative	2 (7.1%)	2 (7.1%)	9 (6.6%)	30 (12.3%)	43 (9.9%)	4
9. 給-gei + verb/noun	2 (7.1%)	3 (10.7%)	30 (22.1%)	47 (19.3%)	82 (18.9%)	2
10. 成-cheng + noun	0 (0%)	1 (3.6%)	1 (0.7%)	8 (3.3%)	10 (2.3%)	8
11. Adv + verb	1 (3.6%)	4 (14.3%)	14 (10.3%)	19 (7.8%)	38 (8.7%)	5

*Participants producing ba sentences: *N* = 97 (age 2;6 = 17; age 3;6 = 15; age 4;6 = 27; and age 5;6 = 38).*

In terms of the ba construction types used by the different age groups, more than half of them (types 1, 2, 3, 8, 9, and 11) were found to emerge at age 2;6, and other three types (types 4, 5, and 10) emerged slightly later in the corpus at age 3;6. Types 7 and 6 occurred the latest, at ages 4;6 and 5;6, respectively. It seemed that while children in the age 2;6 group had a relatively small repertoire of ba constructions, they were capable of using ba sentences in their speech to express meanings regarding different pragmatic contexts, especially a resultative context.

### The Developmental Pattern of Mandarin-Speaking Children’s Acquisition of the ba Construction

**FIGURE 1:**
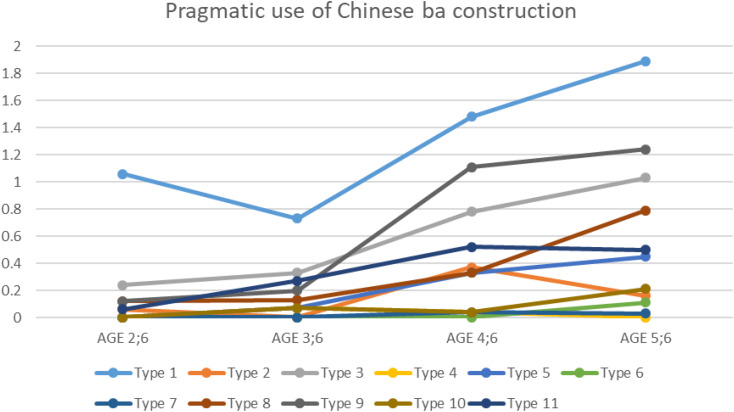
Developmental trend of the pragmatic use of the Chinese ba construction. Note: *x*-axis: Age groups; *y*-axis: Mean value of pragmatic use; participants producing ba sentences: *N* = 97 (age 2;6 = 17; age 3;6 = 15; age 4;6 = 27; and age 5;6 = 38).

Overall, an increasing trend in the use of ba construction was identified in four age groups: 6.4% of the 2-year-olds, 6.4% of the 3-year-olds, 31.3% of the 4-year-olds, and 55.9% of the 5-year-olds, as shown in Table 2. In particular, based on mean values of the participants’ pragmatic use, six types of ba construction (types 1, 9, 3, 8, 5, and 11) were overall found to increase with age, as shown in Figure 1. It seems that age 3;6 marks a turning point, with a noticeable increase in the pragmatic use of ba constructions between age 3;6 and age 5;6. Moreover, a one-way ANOVA on the age effect found that there were significant differences among the four age groups in terms of producing three types of ba construction: type 3 [*F*(3, 93) = 3.895, *p* = 0.011], type 8 [*F*(3, 93) = 3.947, *p* = 0.011], and type 9 [*F*(3, 93) = 4.407, *p* = 0.009]. However, no statistically significant difference was observed in producing the other eight types.

Additionally, regarding the effect of gender on the pragmatic use of ba constructions, the output from a one-way ANOVA showed no statistically significant differences between girls and boys: type 1 [*F*(1, 95) = 0.001, *p* = 0.980], type 2 [*F*(1, 95) = 0.126, *p* = 0.723], type 3 [*F*(1, 95) = 0.154, *p* = 0.696], type 4 [*F*(1, 95) = 0.000, *p* = 0.988], type 5 [*F*(1, 95) = 0.147, *p* = 0.702], type 6 [*F*(1, 95) = 0.000, *p* = 0.983], type 7 [*F*(1, 95) = 2.000, *p* = 0.161], type 8 [*F*(1, 95) = 2.526, *p* = 0.115], type 9 [*F*(1, 95) = 0.861, *p* = 0.356], type 10 [*F*(1, 95) = 0.001, *p* = 0.977], and type 11 [*F*(1, 95) = 0.003, *p* = 0.954] (see Table 3).

**TABLE 3:** The distribution of ba constructions between girls and boys.

Types of ba constructions	Girls (number of pragmatic uses = 217)	Boys (number of pragmatic uses = 218)	Total (number of pragmatic uses = 435)	*P*-value
1. V + resultative verb complement	70	71	141	0.980
2. V + (_一_-yi) + V	7	10	17	0.723
3. V + noun (possessive, person, resultative, and partitive)	36	33	69	0.696
4. V + quantified phrase	1	1	2	0.988
5. V + 了-le	15	12	27	0.702
6. V + 了-le/著-zhe + verb	2	2	4	0.983
7. V + 得-de	0	2	2	0.161
8. V + 在-zai/到-dao + locative	28	15	43	0.115
9. 給-gei + verb/noun	34	48	82	0.356
10. 成-cheng + noun	5	5	10	0.977
11. Adv + verb	19	19	38	0.954

*Participants producing ba sentences: *N* = 97 (girls = 48; boys = 49).*

## Discussion

Using a rich Mandarin Chinese corpus, the present study investigated the acquisition of the ba construction among 168 native Chinese preschool children aged from 2;6 to 5;6. Overall, the results presented above reveal that the participants acquired the ability to produce and use some forms of ba construction from 2 years old and showed mature pragmatics at age four (4;6). Their pragmatic acquisition of the ba construction was also found to be an incremental development process, with a turning point at age 3;6 and a noticeable increase from age 4;6. Beyond generally demonstrating these developmental trends in the children’s linguistic ability, this research has further indicated the impact of age and gender on their production of the ba construction.

Regarding the repertoire of preschoolers’ ba constructions, this research found that in total they produced 11 types of ba construction at 5 years old (age 5;6), which was close or even equal to Mandarin-speaking adults’ relevant pragmatic use (e.g., Liu, 1997). Thus, young children of this age may have reached the requisite level of cognitive development and acquired the ability to express this canonical SVO order in Mandarin Chinese. This is in line with findings reported by previous studies, such as Li (1993); Li et al. (1990), and Yi (2014). Thus, given the uniqueness of the ba construction to Chinese (Tsao, 1987; Xu, 2011), the period around the age of 4;6 may need to be viewed as a critical period to observe Mandarin-speaking children’s language development. In this sense, it will also be necessary to conduct studies of the acquisition of the ba construction among children with specific language impairments (SLI). Relevant studies can provide practical and theoretical evidence to accurately identify children with SLIs, allowing timely and effective interventions to help them (Zeng et al., 2013; Guan, 2016).

Moreover, the data analysis also suggested that “ba + V + resultative verb complement,” “ba + 给-gei + verb/noun,” and “ba + V + noun (possessive, person, resultative, and partitive)” were the three most frequently used ba sentence forms in the corpus, which is in agreement with Li et al. (1990) but differs partially from Wang (2012). Because the ba construction is mainly correlated with telic events and the perfective aspect in Chinese, most of the children’s ba utterances were used to express result states or locations in their daily speech, which are “typically encoded by a resultative or directional verb complement” (Deng et al., 2018, p. 245). For the same reason, it was found that “ba + V + 了-le/着-zhe + verb” was the least used type of ba construction in the corpus; this represented a durative state, such as:

把它塞着煮
Ba it stuff-ASP boil
Push it in (somewhere) to boil

Different from Wang’s (2012) finding, this research showed that the “ba + 给-gei + verb/noun” pattern was widely used in this corpus. One possible explanation behind this difference is that all the participants in the present research were native Mandarin Chinese speakers. According to Zhang (2010), because Mandarin comes from a major group of North Chinese dialects, Mandarin Chinese speakers usually use “给-gei” as a handling or manipulative verb, which inherently agrees with the disposal meaning of the ba construction. In this regard, future studies on the ba construction acquisition should pay more attention to children from different dialectical backgrounds, and comparative research with children from various linguistic groups needs to be conducted to explore whether or not a cross-dialect difference truly exists.

In terms of the developmental pattern of children’s ba constructions, this study indicated that participants from the youngest age group in the corpus (age 2;6) produced six types of ba construction, with a frequency proportion of 6.4%. This showed a slight difference from the production of the age 3;6 group, who produced eight types accounting for 6.4%. However, children at age 4;6 showed an apparent increase in both the pragmatic type (10) and the frequency portion (31.3%) concerning the pragmatic use of ba constructions. This result is congruent with findings from prior studies (e.g., Li et al., 1990; Wang, 2012), which unanimously reported that age 4;6 is a critical period for the development of mature pragmatics in the use of ba constructions. In this sense, it is likely that children have reached a sufficient level of brain, cognitive, and linguistic development by approximately 4 years old, and this level of development now allows them to mentally and practically encode and represent telic events by using different types of ba sentences in their daily life. At the same time, compared to other pragmatic meanings of ba constructions, it was found that preschoolers’ ability to use resultative verb complements and denote the resultative state was at the highest level from a very early age, but the overall frequency proportion of “ba + V + resultative verb complement” in all pragmatic use seemed to decrease as they got older. This was further confirmed by a one-way ANOVA result, which indicated no association between children’s age and their ability to produce this type of ba construction.

Overall, the ba construction requires the verb or verb phrase to be highly resultative or transitive (Li, 1993; Sun, 1995; van der Lee, 2018). The early acquisition of the syntactic, semantic, and verb features of the ba construction in Mandarin Chinese can also be theoretically accounted for in Slobin’s Basic Child Grammar (Slobin, 1985). Based on cross-linguistic findings from many language acquisition studies, Slobin (also see Bavin, 1995) hypothesizes that a Basic Child Grammar, i.e., an innate knowledge of perceiving, storing, and classifying speech input and problem solving strategies, exists and guides children’s language acquisition prior to their first form-function mapping experience of a specific language. The hypothesis further assumes that salient notions of prototypical events and situations play a critical role in the earliest phase of children’s language acquisition. Drawing on cross-linguistic developmental patterns, this is one of the most important temporal perspectives and a valuable theory accounting for how children to observe events and acquire relevant linguistic forms. In addition, the transitive event, like object transfer (e.g., “She gives me a book”), is a salient notion embedded in children’s conceptualizations of prototypical events. The findings concerning ba constructions produced by Mandarin-speaking children indicating a resultative meaning (type 1 and 3) and a transitive meaning (type 9) are aligned with the principles of Slobin’s hypothesis that temporal perspectives and salient notions play an important role in encoding form-meaning mappings and acquiring structured speech during children’s early language acquisition.

In addition, the results from one-way ANOVAs suggested that, with increasing age, children became more competent in using three types of ba construction (types 3, 8, and 9). That is, children’s acquisition of ba construction may have different developmental patterns for different sentence forms. Based on Slobin’s (1985) Basic Child Grammar hypothesis, the significant differences in producing the relevant ba sentences showed that age group differences with respect to pre-structured semantic notions emerged only in a few types of ba constructions. This is good evidence that there may not be a positive correlation between children’s innate linguistic knowledge and age, at least in the present study. According to Li et al. (1990); Liu (1997), and Deng et al. (2018), co-occurring verbs in the ba construction normally play an important role in forming ba predicates, and thus we call for future studies to explore children’s acquisition and use of verbs and their ability to produce ba utterances. Another suggested direction for researchers would be to examine how children use pragmatic approaches to ba sentence production, such as replacing, extending, and changing sentence constituents. Moreover, researchers could focus on the irregular use of ba constructions, since such misuse partly reflects that children under 6 years old may lack a comprehensive understanding of the lexical, syntactic, or pragmatic feature of the ba construction. Relevant knowledge of this issue may help to facilitate children, especially children with specific language impairments, to construct cognitive concepts and produce sentences related to telic events. Furthermore, it was found that there was no relationship between children’s gender and their ability to produce different types of ba construction. In other words, no significant difference regarding innate knowledge of ba constructions emerged between male and female participants. Given its uniqueness to Chinese, with no corresponding syntactic construction in other languages, evidence is needed from other Chinese language-speaking contexts apart from Mandarin. It would be useful for child language researchers to continue investigating gender differences in children’s acquisition of the ba construction and other syntactic structures.

## Conclusion

Using naturalistic language data from the Early Child Mandarin Corpus (Li and Tse, 2011), this study has investigated the development of the ba construction among four age groups of Mandarin-speaking preschoolers. In particular, analysis of these children’s spontaneous utterances suggested that: (1) the Mandarin-speaking children in our corpus produced a repertoire of 11 types of ba construction, and those from the youngest age group (age 2;6) had already developed the ability to produce six types of them; (2) there was an overall increasing trend in ba construction production as children got older, and age 4;6 seemed to be a critical period in terms of the pragmatic use of ba construction; (3) children constructed concepts and denoted the meanings of telic events at a very early age (2;6), and their cognitive and linguistic maturity was close or even equal to adults in terms of producing the ba construction from 5 years old (age 5;6); (4) there was a significant age effect on three types of ba construction (types 3, 8, and 9); and (5) there was no significant association between children’s gender and the frequency of their use of ba constructions.

The present study has some limitations. First, it only involved Mandarin-speaking children, and any generalization of the results to children from other dialect backgrounds in China needs to be undertaken with caution. Expanding the sample size and including children from different dialect backgrounds may help mitigate this problem to some extent. Second, this study used natural utterances from a free-play session, and the children were accompanied only by toys and a peer. In other words, they may not have produced as many target language utterances in this situation as they do in actual everyday contexts. Third, while the language data were collected from four age groups of children, the short-term nature of the data collection limited us from any examination of children’s developmental trajectory in relation to the ba construction. It would be helpful to adopt a longitudinal design to map detailed changes or progress in acquiring the ba construction over time (Gong et al., 2020c,d). In addition, as can be seen from this research, because the participants’ ability to produce different types of ba construction developed quickly from 2;6 to 5;6, the age gap between each participant group may need to be narrowed down further in future studies.

Despite these limitations, however, we believe that the findings of this research offer a contribution to furthering our understanding of the development of the ba construction in childhood Mandarin Chinese. This research also calls for more attention to the language development of children with different linguistic and cognitive backgrounds (Liang et al., 2019).

## Data Availability Statement

The source data of this research has been publicly shared already through the OSF (Reference number: DOI: 10.17605/OSF.IO/EJFN4; Link: https://osf.io/3as4q).

## Author Contributions

YG and LT: formal analysis. YG: writing-original draft. LT and YG: writing-review and editing. LT and YG: funding acquisition. YG and LT: project administration. Both authors contributed to the article and approved the submitted version.

## Conflict of Interest

The authors declare that the research was conducted in the absence of any commercial or financial relationships that could be construed as a potential conflict of interest.
