# Supplementary material for: A Corpus-Based Study on the Pragmatic Use of the ba Construction in Early Childhood Mandarin Chinese
Source: Front Psychol. 2021 Jan 15;11:607818. doi: 10.3389/fpsyg.2020.607818 (PMC7874079; doi:10.3389/fpsyg.2020.607818)
Supplement: Supplementary file 1 [file Table_1.pdf]

Appendix 1: 9-type framework for ba constructions proposed by Liu (1997)

Category	Example
1) V + resultative verb complement	你把问题看清楚<br>Ni ba wenti kan qingchu<br>You ba question read clear<br>You read the questions so that they are clear
2) V + 得-de (resultative)	他把我哭得心烦<br>Ta ba wo ku de xinfan<br>He ba I cry DE heart-disturbed<br>He cried so much that I became disturbed
3) V + retained object	他把大门上了锁<br>Ta ba damen shang-le suo<br>He ba gate put-on-ASP lock<br>He locked the gate
4) V + perfective marker 了-le	他把苹果吃了<br>Ta ba pingguo chi-le<br>He ba apple eat-ASP<br>He ate the apple
5) V + PP (dative or locative)	我把字典借给老王<br>Wo ba zidian jie gei Laowang<br>I ba dictionary lend to Laowang<br>I lent the dictionary to Laowang
6) V + quantified phrase	他把电影看了两遍<br>Ta ba dianying kan-le liangbian<br>He ba movie watch-ASP twice<br>He watched the movie twice
7) V + 一-yi + V (the tentative construction)	请你把信看一看<br>Qing ni ba xin kan-yi-kan<br>Please you ba letter read-one-read<br>Please read the letter a little
8) V + durative marker 着-zhe	把证件带着<br>Ba zhengjian dai-zhe<br>ba ID carry-ASP<br>Carry your ID
9) Adv + verb	我把桌子往屋里搬<br>Wo ba zhuozi wang wuli ban<br>I ba table towards room-in move<br>I was moving the table into the room
