# Supplementary material for: A Corpus-Based Study on the Pragmatic Use of the ba Construction in Early Childhood Mandarin Chinese
Source: Front Psychol. 2021 Jan 15;11:607818. doi: 10.3389/fpsyg.2020.607818 (PMC7874079; doi:10.3389/fpsyg.2020.607818)
Supplement: Supplementary file 2 [file Table_2.pdf]

Appendix 2: 6-category framework for ba constructions proposed by Wang (2012)

Category	Example
1) V + complement	
1a) V + directional verb	把這個打開<br>Ba zhege dakai<br>Ba this open<br>Open this
1b) V + verb	把飯都吃完<br>Ba fan dou chi wan<br>Ba meal all eat finish<br>Finish the meal
1c) V + adjective	把胳膊摔壞了<br>Ba gebo shuai huai-le<br>Ba arm fall down broken-ASP<br>Broke the arm
1d) V + quantitative phrase	把它吓一跳<br>Ba ta xia yi-tiao<br>Ba it scare one-jump<br>It is so scared that it leaps
2) V + 在-zai/到-dao + locative	把它放在嘴里<br>Ba ta fang zai zuili<br>Ba it put ZAI mouth-in<br>Put it into mouth
3) V + (一-yi) + verb	
3a) V + 一-yi + verb	把它洗一洗<br>Ba ta xi-yi-xi<br>Ba it wash-one-wash<br>Wash it a little
3b) V + verb reduplication	把脚拍拍<br>Ba jiao pai-pai<br>Ba feet pat-pat<br>Pat your feet
4) V + 了-le/着-zhe	把这个扔了<br>Ba zhege reng-le<br>Ba this dump-ASP<br>Dump this<br><br>把土拿着<br>Ba tu na-zhe<br>Ba soil hold-ASP<br>Hold the soil
5) V + noun	
5a) V + possessive noun	把它剥皮<br>Ba ta bo pi

	Ba it peel skin<br>Peel it
5b) V + 成-cheng + noun	把它弄成面包<br>Ba ta nong cheng mianbao<br>Ba it make CHENG bread<br>Make it into bread
6) 给-gei	
6a) 给-gei + noun	把那个给我<br>Ba nage gei wo<br>Ba that GEI me<br>Give that to me
6b) 给-gei + noun + verb	把这个给它标上<br>Ba zhege gei ta biao shang<br>Ba this GEI it mark up<br>Mark this on it
6c) 给-gei + verb	把这个奥特曼给粘上<br>Ba zhege aoteman gei nian shang<br>Ba this Ultraman GEI glue up<br>Glue this Ultraman together
