# Supplementary material for: A Corpus-Based Study on the Pragmatic Use of the ba Construction in Early Childhood Mandarin Chinese
Source: Front Psychol. 2021 Jan 15;11:607818. doi: 10.3389/fpsyg.2020.607818 (PMC7874079; doi:10.3389/fpsyg.2020.607818)
Supplement: Supplementary file 3 [file Table_3.pdf]

Appendix 3: 9-type framework for ba constructions proposed by Li et al. (1990)

Category	Example
1a) V + verb/adjective	我把球甩掉<br>Wo ba qiu shuai diao<br>I ba ball throw away<br>I threw away the ball
1b) V + directional verb	把这个手套搞下来<br>Ba zhege shoutao gao xialai<br>Ba this glove get down<br>Take off this glove
2) V + 在-zai/到-dao + locative	把我孩子拖到家里去了<br>Ba wo haizi tuo dao jiali qu-le<br>Ba my kid drag DAO home-in go-ASP<br>Drag my kid home
3a) Adv + verb	把这个衣服先脱掉<br>Ba zhege yifu xian tuo diao<br>Ba this clothes first take off<br>Take off the clothes first
3b) 一-yi + verb	他把火车一放<br>Ta ba huoche yi fang<br>He ba train one put<br>He put down the train
3c) 給-gei + verb	把我给累死了<br>Ba wo gei lei si-le<br>Ba me GEI exhaust die-ASP<br>Exhaust me to death
3d) prepositional phrase + verb	把甘蔗用凉水搞搞<br>Ba ganzhe yong liangshui gao gao<br>Ba sugarcane use cool water get get<br>Soak the sugarcane with cool water
4a) V + 了-le	大灰狼把我们吃了<br>Da huilang ba women chi-le<br>Big grey wolf ba us eat-ASP<br>The big grey wolf ate us
4b) V + 着-zhe	把两个都拿着<br>Ba liangge dou na-zhe<br>Ba two both hold-ASP<br>Hold both of them
5a) V + possessive noun	把嘴扎流血<br>Ba zui zha liuxue<br>Ba mouth stab bleed<br>Stab the mouth to bleed
5b) V + noun (person)	把我的纸给我<br>Ba wode zhi gei wo

	Ba my paper give me<br>Give my paper to me
5c) V + resultative noun	把这个染上颜色<br>Ba zhege ran shang yanse<br>Ba this dye up color<br>Dye this
5d) V + partitive noun	把头砍半块<br>Ba tou kan bankuai<br>Ba head cut half pieces<br>Cut the head into halves
6) V + verb reduplication	把车修一修<br>Ba che xiu-yi-xiu<br>Ba car fix-one-fix<br>Fix the car a little
7) V + 得-de + modal phrase	把你戳得像个什么样子<br>Ba ni chuo de xiangge shenme yangzi<br>Ba you poke DE like what look<br>Poke you into such a look
8) V + verb + classifier	把话都说一遍<br>Ba hua dou shuo yi bian<br>Ba word all say one time<br>Say all the words once
9) 当-dang/搞成-gaocheng + noun + (verb)	把小白兔当点心吃<br>Ba xiao baitu dang dianxin chi<br>Ba small white rabbit DANG snack eat<br>Eat the small white rabbit like a snack<br><br>把沙搞成正方形<br>Ba sha gaocheng zhengfangxing<br>Ba sand GAOCHENG square<br>Make the sand into a square
