# Supplementary material for: A Corpus-Based Study on the Pragmatic Use of the ba Construction in Early Childhood Mandarin Chinese
Source: Front Psychol. 2021 Jan 15;11:607818. doi: 10.3389/fpsyg.2020.607818 (PMC7874079; doi:10.3389/fpsyg.2020.607818)
Supplement: Supplementary file 4 [file Table_4.pdf]

Appendix 4: 13-case framework for ba constructions proposed by Lü (1984)

Category	Example
Extra objects	
1) V + partitive object	把一个南京城走了大半个<br>Ba yige nanjingcheng zou-le da bange<br>Ba one Nanjing city go-ASP big half<br>Walk through half of Nanjing City
2) V + quantitative object (verb/verb reduplication)	把两手拍了一下<br>Ba liang shou pai-le yixia<br>Ba two hands clap-ASP once<br>Clap both hands<br><br>把脸擦擦<br>Ba lian ca ca<br>Ba face wipe wipe<br>Wipe the face
3) V + retained object	把大门上了大闩<br>Ba damen shang-le da shuan<br>Ba gate up-ASP big bolt<br>Bolt the gate
Complements	
4) V + recipient (with or without 给-gei)	把这等的机密大事告诉了你<br>Ba zhe deng de jimi dashi gaosu-le ni<br>Ba this level de classified matter tell-ASP you<br>Tell you about this classified matter<br><br>把帽子摘了递给华忠<br>Ba maozi zhai-le di gei Huazhong<br>Ba hat take off-ASP pass GEI Huazhong<br>Take off the hat and pass it to Huazhong
5) V + complement of place	把碟子挪在跟前<br>Ba diezi nuo zai genqian<br>Ba plate move to front<br>Move the plate to the front
6) V + complements of direction and aspect	把茶点来<br>Ba cha dian lai<br>Ba tea order come<br>Order some tea and bring it here
Complements of result	
7) without 得-de	把他逼走了<br>Ba ta bi zou-le<br>Ba him drive go-ASP<br>Drive him away
8) with 得-de	把话说得越坚决越好

	Ba hua shuo de yue jianju yue hao<br>Ba word say DE more determined better<br>The more assertively the word is said, the better it is
9) special cases (causative or means)	把我羞哭了<br>Ba wo xiu ku-le<br>Ba me ashamed cry-ASP<br>Make me so ashamed that I burst into tears<br><br>把手绢儿哭湿<br>Ba shoujuan-er ku shi<br>Ba handkerchief cry wet<br>Cry and make the handkerchief wet
10) negative result	把凤丫头病了<br>Ba Feng yatou bing-le<br>Ba Feng yatou sick-ASP<br>Make Feng yatou ill
11) 一-yi + verb	把桃儿一放<br>Ba tao-er yi fang<br>Ba tao-er one put<br>Put down the peach
12) 都-dou + verb	把一钟酒都干了<br>Ba yizhong jiu dou gan-le<br>Ba one wine all dry-ASP<br>Drink up the bottle of wine
13) other cases	把箱子一齐打开<br>Ba xiangzi yiqi dakai<br>Ba boxes together open<br>Open the boxes together
